# Cost-efficient long-read trio-barcoded adaptive sequencing improves rare disease diagnosis

**DOI:** 10.1038/s41467-026-77535-8

**Published:** 2026-09-04

**Authors:** Yilei Fu, Adam C. English, Luis F. Paulin, Shalini N. Jhangiani, Nikhita Gogate, George Weissenberger, Vanessa Vee, Han Yi, Heer H. Mehta, Donna M. Muzny, Richard A. Gibbs, Jennifer E. Posey, Daniel G. Calame, Fritz J. Sedlazeck

**Affiliations:** 1 Human Genome Sequencing Center, Baylor College of Medicine, Houston, TX USA; 2 Department of Molecular and Human Genetics, Baylor College of Medicine, Houston, TX USA; 3 Departments of Pediatrics and Medicine, Columbia University Vagelos College of Physicians and Surgeons, New York, NY USA; 4 Section of Pediatric Neurology and Developmental Neurosciences, Department of Pediatrics, Baylor College of Medicine, Houston, TX USA; 5 Texas Children’s Hospital, Houston, TX USA; 6 Department of Computer Science, Rice University, Houston, TX USA

**Keywords:** Genome informatics, Biotechnology, Genetics

## Abstract

Rare diseases often remain unsolved because causal genetic changes can be complex and thus missed by standard sequencing or difficult to prioritize. Long-read sequencing can reveal structural variants, repeat expansions, DNA methylation and inherited haplotypes, but trio sequencing of an affected child and both parents remains costly. Here we show that phenotype-driven Trio-barcoded Oxford Nanopore Adaptive Sequencing (TBAS) enables cost-efficient long-read analysis of rare-disease trios on one flow cell. TBAS workflow uses clinical features to select broad disease-gene panels, barcodes all three family members and enriches these regions during sequencing rather than targeting a known causal locus. In benchmark regions, TBAS increased coverage and accurately detected small variants, structural variants, tandem repeat expansions, methylation and read-backed phasing, while reducing estimated sequencing consumable costs to 32.2% of conventional three-flow-cell trio long-read sequencing. Across 13 trios, TBAS recovered all five known diagnoses and prioritized candidates in five of eight unresolved cases.

## Abstract (web summary)

Here the authors present Phenotype-driven Trio-barcoded Adaptive Sequencing (TBAS) that enables cost-efficient long-read sequencing of rare-disease trios on a single flow cell, detecting diverse genetic variants while cutting costs to 32% of standard trio sequencing.

## Introduction

Rare diseases (RDs) are actually quite common, collectively affecting one out of twenty people worldwide^1,2^. Many efforts, such as the GREGoR (Genomics Research to Elucidate the Genetics of Rare Diseases) Consortium, aim to improve rare disease detection and interpretation, but significant challenges remain^3^. Advances in sequencing technologies, bioinformatics, and molecular profiling now allow earlier and more accurate diagnoses, which is crucial given that many rare diseases are severe, progressive, and often undiagnosed. The introduction of long-read sequencing (LRS) has advanced rare disease research by capturing structural variants (SV) and tandem repeats (TR) that were previously inaccessible, and by delivering long-range haplotypes and direct methylation profiles in trio-based analyses^4,5^. Trio sequencing is indispensable for rare disease diagnosis because it reveals de novo variants and clarifies variant phasing^6–8^. However, the cost of three separate long-read sequencing runs hinders scaling and limits clinical adoption^9^. Simultaneously, the annotation of genome-wide variants remains challenging^10^. This is often aided by gene lists of hundreds of genes associated with phenotypic categories, for example epilepsy or intellectual disability. These gene lists inform variant, gene, and locus prioritization that aid in interpretation.

**Fig. 1:**
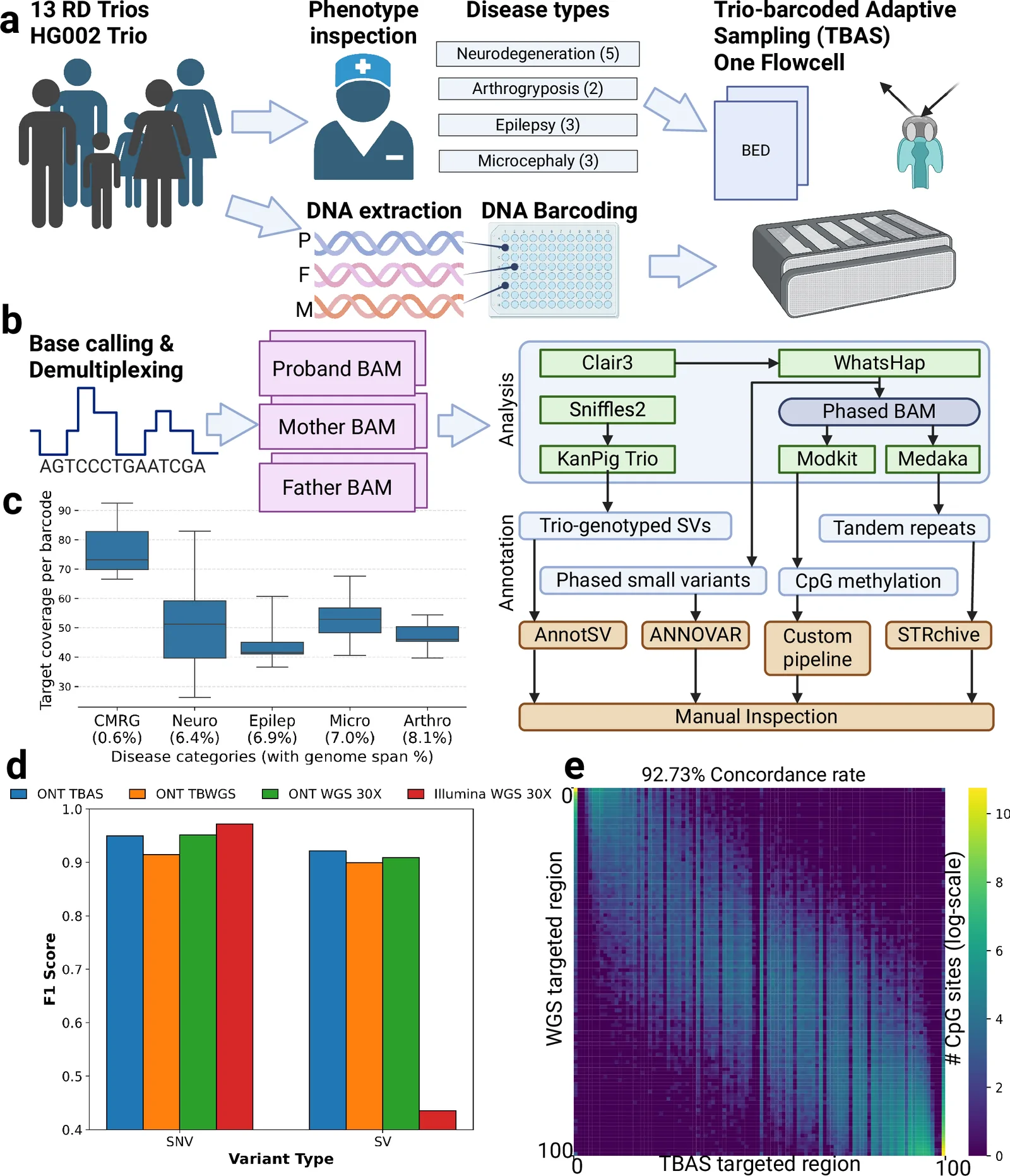
Sequencing strategy, analysis pipeline, and benchmarking statistics of trio-barcoded adaptive sequencing. **a** Sequencing strategy, sample selection, phenotype annotation, DNA preparation and adaptive sequencing. The HG002 family was sequenced over CMRG benchmark regions. Rare disease (RD) trios (*N* = 13) were sequenced over their respective phenotype-linked gene lists. DNA was extracted and barcoded per individual and ONT adaptive sequencing was performed using one flow cell per trio configured with the corresponding phenotype-linked target panel. **b** Variant analysis and annotation pipeline. Basecalling and demultiplexing produces individual BAM files separately for each sequencing experiment before performing variant calling and annotation. **c** Sequencing coverage achieved over targeted regions by disease category. **d** Variant calling performance benchmarking on targeted CMRG regions. **e** DNA methylation concordance map of each CpG on the targeted regions between TBAS and WGS. Created in BioRender. Fu, Y. (2026) https://BioRender.com/cmp8qwz.

In this study, we present Trio-barcoded ONT Adaptive Sequencing (TBAS) workflow, a cost-efficient long-read strategy for unresolved rare-disease trios that uses broad phenotype-informed disease-gene panels, rather than a known causal locus, to guide adaptive enrichment of barcoded family samples on a single ONT PromethION or P2 flow cell (Fig. 1a)^11–13^. Using public ONT list prices, TBAS workflow reduces estimated ONT sequencing consumable costs to 32.2% of a conventional three-flow-cell ONT long-read trio WGS strategy (Methods). while simultaneously enriching coverage to average 49x (~2 fold compared to ONT WGS) across disease-relevant genomic regions tailored to the patient’s phenotype. By directing long-read sequencing to regions most relevant to patient phenotypes, our strategy (Fig. 1b) enables accurate detection of de novo and inherited variants, providing a path toward cost-effective, clinically deployable rare disease diagnostics using long-read sequencing at scale.

## Results

We first assessed the performance of the TBAS workflow on the HG002, HG003 & HG004 family, targeting the GIAB Challenging Medically Relevant Gene benchmark (CMRG) regions with 395 genes spanning 0.5% of the genome^14^. This experiment was used as a technical benchmark of coverage, variant calling, phasing and methylation performance, not as a rare-disease discovery experiment. For comparison, the barcoded and pooled trio was also sequenced using one PromethION flow without adaptive sequencing. TBAS targeted regions averaged ~77x sequencing coverage (Fig. 1c, Supplementary Fig. 1), while trio-barcoded ONT whole-genome sequencing (TBWGS) reached only ~12x (Supplementary Data 1). For analysis, we designed a comprehensive pipeline to identify and phase all genetic variants and DNA methylation. Subsequently, automatic variant annotation and prioritisation were performed (methods; Fig. 1b). This reproducible workflow integrates established long-read tools for variant calling, phasing, methylation analysis, tandem repeat analysis and variant prioritization, and is available at https://github.com/Fu-Yilei/TBAS_pipeline

The CMRG benchmark regions measured TBAS workflow performance with F1-scores of 94.95% for small variants and 92.19% for SV (Supplementary Data 2), exceeding the performance of TBWGS at 91.74% and 89.95%, respectively. This is due to the increased sequencing coverage on targeted regions from TBAS workflow, which led to the identification of seven additional true positive SVs and two fewer false positive SVs. For comparison, publicly available HG002 datasets sequenced with Illumina and ONT at ~30X coverage^15^ were analyzed. TBAS workflow performance was comparable to Illumina short-read derived small variants called by deepvariant^16^ (F1-score 97.16%, +2.19% compared to TBAS) and closely matched ONT WGS (F1-score 95.12%, +0.17% compared to TBAS). For SV, TBAS workflow substantially outperformed Illumina WGS (F1-score 43.56%, −48.63% compared to TBAS) and was similar to ONT WGS at 30X coverage (90.91%, −1.28% compared to TBAS; Fig. 1d). Furthermore, TBAS workflow achieved long-read based phasing for 99.5% of all variants. Lastly, DNA methylation pileups from TBAS workflow showed a high concordance rate of 92.73% relative to 30X HG002 public ONT WGS (Fig. 1e).

During adaptive sequencing, the first 400–500 bases of reads are sequenced before being recognized as on- or off-target, with off-target reads then being rejected for further sequencing^13^. These rejected reads can still be analyzed, albeit with some limitations in coverage and read length. The total read set (on-target and rejected) captured by the TBAS sequencing experiment produced genome-wide coverage of 6.07x with read length N50 of 531, 85.72% recall and 81.13% precision for SNV discovery, and 23.08% recall and 84.79% precision of SV calling (Supplementary Data 2). Supplementary Figs. 3, 4 show some examples of false positive and negative SV which were impacted by alignments. Genome-wide DNA methylation analysis had a WGS concordance rate of 78.58%. This shows that while we observed the expected reduced performance of off-target regions compared to on-target, it’s still possible to obtain a considerable amount of off-target variants and methylation when needed.

Given the successful demonstration of our approach, the TBAS workflow was used to analyze 13 rare disease trios falling into four phenotypic classes: epilepsy, arthrogryposis, microcephaly and neurodegeneration. Each phenotype class had a set of 1,662 to 2,582 associated genes identified through the medical literature and Mendelian disease databases such as OMIM (Supplementary Data 3). These gene lists spanned between 6.4% and 8.1% of GRCh38. Despite the larger target gene lists (Fig. 1c), the average sequencing depth achieved by TBAS on targeted regions was ~49x (Supplementary Data 1). Among the 13 trios, 5 were previously solved^17–20^ and 8 were previously undiagnosed after clinical exome and whole genome Illumina sequencing (Supplementary Data 4). TBAS and our pipeline identified the causative variant as the top candidate in 5 out of 5 solved cases (Supplementary Section 3) and provided potentially causative variants in 5 out of 8 unsolved cases (Supplementary Data 5 and 6), resulting in a 76.9% potential solve rate across the 13 trios.

**Fig. 2:**
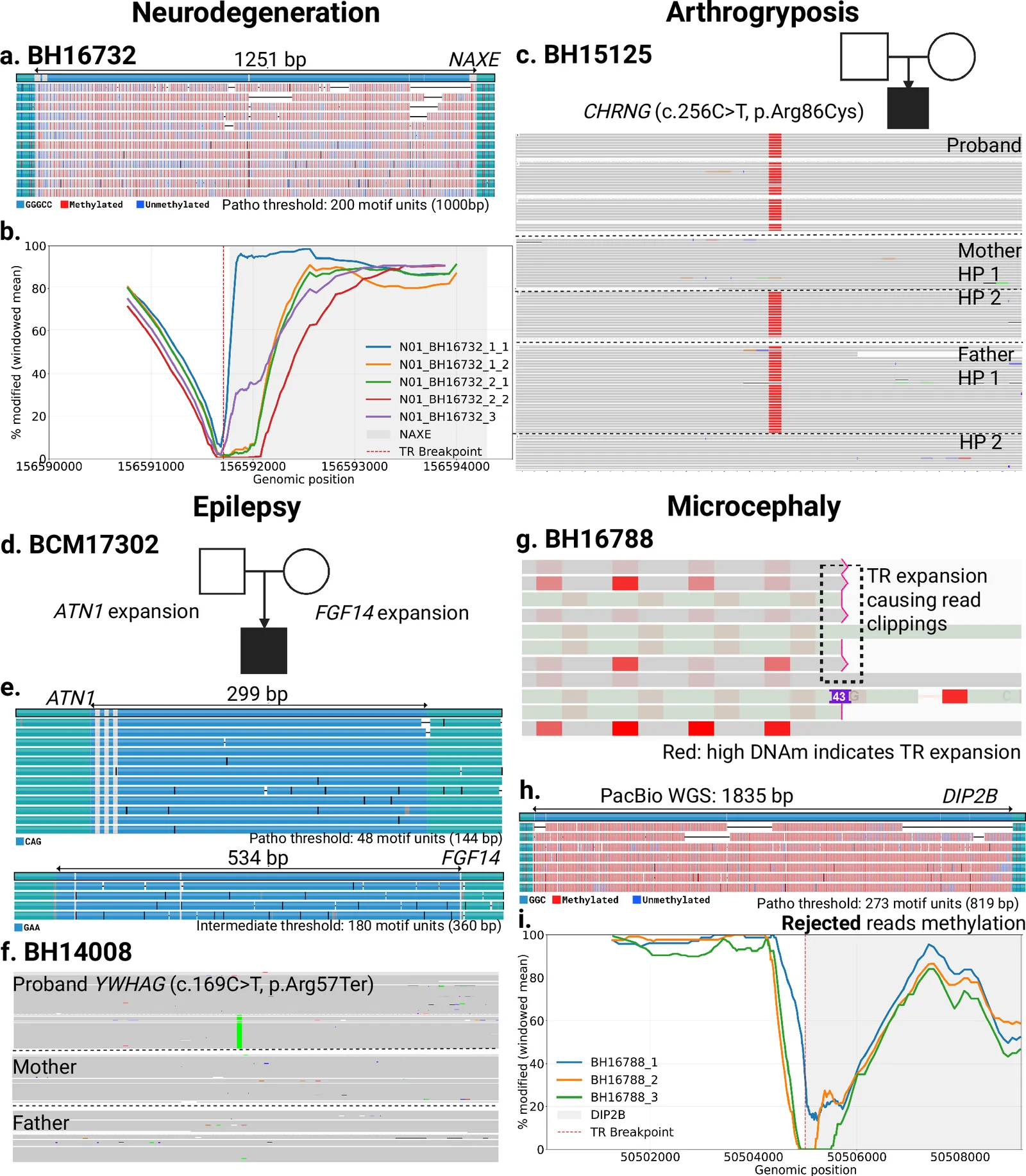
Analysis of selected RD cases. The middle circle indicates each disease category. **a** The expanded allele of NAXE in BH16732_1. **b** The DNA methylation profiling around the NAXE repeat expansion shows a hypermethylation in the proband’s expanded allele and an allele-specific DNA methylation in the father. 1_1, 1_2: Haplotype 1 and 2 of BH16732 proband, 2_1, 2_2: Haplotype 1 and 2 of BH16732 mother, 3: unphased allele specific methylation of BH16732 father. **c** Homozygous SNV on an unsolved case BH15125. The IGV screenshot shows the proband has the homozygous SNV while both parents are heterozygous. **d** The inheritance of two repeat expansions in BCM17302_1. **e** The BCM17302 proband’s expanded alleles of ATN1 and FGF14. **f** de novo YWHAG SNV discovered in BH14008_1 **g** DIP2B repeat expansion leads to the read clipping of the rejected reads and hypermethylation in those rejected reads. **h** PacBio WGS profiled proband’s expanded allele. **i** Hypermethylation is detected in both proband and mother in rejected reads. Created in BioRender. Fu, Y. (2026) https://BioRender.com/cmp8qwz.

Based on the patient phenotype, 1,704 genes were selected to help resolve 5 examples of neurodegenerative phenotype class trios. On average 235,453 SNV and 2,173 SV were identified per-individual. Analysis of the TBAS-generated data prioritized the known causative variants in all previously solved cases and identified one strong candidate in the three previously unsolved cases (Supplementary 2.1). Among the cases analyzed, the pipeline correctly ranked *CLP1* (NM_006831.3):c.419 G > A, p.Arg140His homozygous variant in two previously solved cases (Supplementary 2.1.1) as the highest rank causative variants. In the case of BH12925_1, previous exome analysis prioritized a homozygous variant in candidate disease gene *KCNJ14* (NM_013348.4:c.643 C > G; p.L215V)^20^; however, no additional cases supporting this gene-disease association have been described. Despite *KCNJ14* being absent from the targeted gene list, TBAS workflow recovered this variant based on a priori knowledge from the paired exome analysis via rejected-read SNV calling. This illustrates that rejected reads can provide limited off-target support when a locus is specifically queried, although such off-target signal is not a substitute for including a candidate locus in the adaptive sampling panel (Supplementary 2.1.2). However, a better disease gene panel could be applied here to retrieve the high-quality disease causing SNP. In the previously unsolved case BH16732, a tandem repeat expansion in *NAXE* was identified as a strong causative candidate. The proband carried a de novo expansion of ~250 repeat motifs, compared to ≤114 motifs on both parental alleles (Fig. 2a, Supplementary 2.1.3, Supplementary Data 6). This expansion was previously not detected by Illumina short-read sequencing, highlighting the value of the long-read component of TBAS workflow for resolving repeat expansions that are difficult to assess with short-read sequencing. This tandem repeat was also detected by our SV discovery pipeline as a de novo 1074 bp insertion in the proband. Notably, *NAXE* repeat expansions are associated with local hypermethylation^21^, which we were able to profile around the read alignment breakpoint of the expansion, revealing methylation alterations consistent with a single previously reported case and thus suggestive of pathogenicity (Fig. 2b). These findings highlight the capability of long reads generated from the TBAS approach in resolving pathogenic repeat expansions and identifying potentially pathogenic variants of all types in rare disease cases.

For the arthrogryposis phenotype class, phenotype group, 2582 genes were selected and TBAS paired with the bioinformatic approach, identified an average of 314,131 SNV and 3041 SV across trios. The top candidate variant for each trio was the previously solved cases’ known variant and the most likely causative variant of both unsolved cases (see Supplementary 2.2 for each case). In one unsolved case, BH15125_1, the analysis of TBAS data identified two homozygous variants: *CHRNG*:c.256 C > T (p.Arg86Cys) and *FBN1*:c.2582 G > A (p.Arg861Gln) (Fig. 2c). The *CHRNG* variant is a known pathogenic allele consistent with the proband’s phenotype (Supplementary 2.2.1) and was previously reported in ClinVar by Pehlivan et al.^22^. The *FBN1* variant alone does not explain the phenotype and is classified as a variant of uncertain significance in ClinVar but may be contributing to the patient phenotype through multi-locus pathogenic variation^20^. In another unsolved case (BH15126_1), TBAS and its paired bioinformatic pipeline identified a homozygous frameshift variant in *CHST14* (ENST00000306243.7:c.145del; p.Val49Ter). Variants in *CHST14* are known to cause musculocontractural Ehlers-Danlos syndrome, a diagnosis consistent with the proband’s clinical presentation (Supplementary 2.2.2). These findings were later confirmed on reassessment of exome sequencing data.

Next, we examined the epilepsy phenotype class where TBAS workflow was used to sequence three trios focusing on 1662 genes in total (Supplementary 2.3). Across the trios we found on average 241,217 SNV and 2172 SV in the targeted regions. In one unsolved case (BCM17302_1), we detected two heterozygous tandem repeat expansions in the proband, located in *ATN1* and *FGF14* (Fig. 2d, Supplementary Data 6). The expanded *ATN1* allele (100 motifs) was inherited from the father (85 motifs) and reached the pathogenic threshold (48 motifs) in both the father and the proband (Fig. 2e). Thus, we suspected that this is an incomplete penetrance that was consistent with father’s moderately expanded repeat. The *FGF14* expansion was inherited from the mother and remained at an intermediate level in both her and the proband. This case illustrates the value of using long-read tandem repeat sizing in trio inheritance analysis. This allele was subsequently validated with PacBio WGS (Fig. 2e). The pathogenic *ATN1* expansion is consistent with the proband’s clinical presentation. While *FGF14* expansions are most commonly linked to late-onset cerebellar ataxia^23^ and do not fully explain the proband’s epilepsy, it nevertheless might contribute to the phenotype (Supplementary 2.3.1). The TBAS analysis pipeline ranked SNVs as the top candidates in two solved cases: a de novo variant in *YWHAG* (NM_012479.4):c.169 C > T (p.Arg57Cys) in BH14008^17^ (Fig. 2f); a homozygous variant of BH14010 in *MED27* (ENST00000292035.10):c.839 C > T (p.Pro280Leu)^18^ (Supplementary Data 5).

Lastly, we further investigated a solved and two unsolved trios associated with microcephaly and 1769 genes selected based on the phenotype and used as the adaptive sampling target panel of the TBAS workflow (Supplementary 2.4). On average, we identified 254,297 SNV and 2468 SV which included the previously identified variants for each solved case, as well as candidate variants for a previously unsolved case. The one previously solved case (BH14573) contained a homozygous missense substitution in exon 16 of *AFG3L2* (NM_006796.3:c.2135 C > T; p.Thr712Ile). Although this case has been attributed to a candidate X-linked gene, *PPP1R3F*^19^, the *AFG3L2* variant provides a strong molecular and clinical fit (Supplementary 2.4.1). The *PPP1R3F* SNP was also discovered in the TBAS data. Its association with developmental delay, microcephaly, abnormal movements, brain atrophy, and deep white-matter changes aligns closely with recent phenotypic expansions of the AFG3L2 disease spectrum^24^. The variant was confirmed by both PacBio whole-genome and Illumina exome sequencing, supporting its role as the likely causal allele. For the case BH16788, an internal review based on Illumina sequencing suggested a DIP2B repeat expansion. Although the *DIP2B* gene fell outside our targeted regions, TBAS data still allowed observed read support and clear methylation gains (Fig. 2g) caused by the repeat expansion^25^ at the locus in both the proband and her mother (Fig. 2h, i). Notably, while the mother harbors the expansion and shows even stronger methylation changes, she remains clinically unaffected, highlighting unresolved questions about penetrance and the pathogenic threshold of DIP2B expansions^26^. This was further confirmed by subsequent PacBio WGS analysis. Neither TBAS workflow, PacBio nor Illumina found any other variants that could be classified as pathogenic or likely pathogenic matching the disease phenotype. This result shows that rejected reads can provide useful off-target support in selected cases, although off-target analysis remains lower coverage and less sensitive than targeted or whole-genome sequencing. For another unsolved proband BH14640, a homozygous missense variant was identified in *UNC80*, which is currently classified as a variant of uncertain significance. After expert clinician review of this variant, it remains uncertain if this is the causative variant. Additional functional studies and detailed phenotypic correlation will be necessary to clarify its pathogenic relevance (Supplementary 2.4.3). Together, these three cases within the microcephaly cohort underscore the capability of TBAS workflow to comprehensively detect homozygous single-nucleotide variants, even refining or correcting previously considered solved cases.

## Discussion

The TBAS workflow provides a cost-efficient and comprehensive strategy for phenotype-guided long-read trio analysis in rare disease. Across 13 GREGoR trios, TBAS recovered the known candidate variant in all five previously solved cases and prioritized likely causative or clinically relevant candidates in five of eight previously unresolved cases, resulting in a 76.9% potential solve rate. Across the previously unresolved cases, TBAS contributed in two complementary ways. For repeat expansion cases, especially *NAXE* and *ATN1*, the long-read data enabled direct allele sizing, inheritance assessment and, in the *NAXE* case, methylation support around the expanded allele. In other cases, the TBAS workflow highlighted and reported clinically relevant SNV or indel candidates that had not been prioritized during earlier clinical review. Retrospective inspection of prior short-read or exome data showed that some of these variants were technically present, but had not been recognized as leading candidates before TBAS analysis. Thus, the added value of TBAS was not limited to detecting variants absent from short-read data. It also provided independent trio-based prioritization and molecular interpretation across variant classes. The accompanying variant calling and annotation pipeline includes variant prioritizations to enable widespread use of this approach. Furthermore, the increased coverage of TBAS would allow for the detection of mosaic variants, although we couldn’t demonstrate this in our cases. Unlike hybrid capture, adaptive sequencing still provides partial access to off-target regions, offering additional contextual information, though at lower coverage and reduced variant-calling sensitivity. However, very small target panels (e.g., 0.6% in HG002) limit enrichment efficiency and could lead to imbalanced barcode coverage, illustrating that adaptive sequencing is most effective when applied to moderately sized, phenotype-informed gene sets. In practice, we have found out the optimal DNA fragment length is 10-12 kbp long and the optimal reference genome span is 5–10% (Methods). Together, these results establish TBAS as a scalable, cost-efficient, phenotype-guided long-read sequencing framework for rare-disease trio analysis.

## Methods

### Consent agreements

All individuals or their guardians provided written informed consent for genomic studies and publication of clinical history under Baylor College of Medicine Institutional Review Board approved protocol H-29697. All individual IDs throughout this work are anonymized.

### Sample selection

All samples in this study were obtained through the GREGoR consortium and had been previously reviewed by consortium clinicians using other established genomic technologies. The only information available to us at the time of analysis was that these cases remained genetically unresolved and their disease phenotypes. Sample selection was restricted to four broad disease categories because the clinician involved in the downstream manual variant inspection has specific expertise in these areas, to ensure that candidate variants could be reviewed by a clinician with relevant disease-area expertise. These disease categories also matched the phenotype-linked panels used for adaptive sampling.

Beyond this high-level disease categorization, no additional selection criteria were applied. In particular, there was no prioritization based on phenotype severity, family structure, or prior diagnostic hypotheses. The sole inclusion criterion was that the cases had not been solved by prior clinical evaluation within the consortium. Before TBAS, some cases had been evaluated using narrower phenotype-specific clinical panels (i.e., solved cases in the manuscript). For this study, we used broader phenotype-category panels so that four standardized target sets could cover the selected disease groups.

### Adaptive sequencing

Genomic DNA was diluted to 30 ng/µl. Starting with 1500 ng for each sample, DNA was sheared using g-tubes (Covaris 520079) to achieve an average size of ~10 kb. The sheared DNA was size-selected on the PippinHT instrument (Sage Science) using the 6–10 kb High-Pass definition with a minimum size selection threshold of 6 kb. Libraries for ONT were prepared using the Native Barcoding Kit 96 V14 (SQK-NBD114.96) wherein each trio was barcoded and pooled into 1 library. Libraries were sequenced on the ONT PromethION 24 device using R10.4.1 flow cells with the adaptive sampling option enabled.

### TBAS costs analysis

Cost estimates were calculated using publicly available ONT list prices for sequencing consumables at the time of analysis. These estimates include flow cells and ONT library/barcoding reagents, but exclude labor, equipment depreciation, negotiated discounts, sample extraction, QC, size selection, shipping, taxes, third-party reagents and institutional overhead.

TBAS trio on one PromethION flow cell:

- 1 PromethION flow cell: $1040
- 1 Native Barcoding Kit reaction for the trio: $79.17
- Total ONT sequencing consumables: $1119.17 per trio

Conventional ONT long-read WGS trio as three separate single-sample runs:

- 3 PromethION flow cells: 3 × $1040 = $3120
- 3 Ligation Sequencing Kit reactions: 3 × $120 = $360
- Total ONT sequencing consumables: $3480 per trio

TBAS consumable cost = $1119.17 / $3480 = 32.2% of the conventional three-flow-cell ONT trio WGS strategy. It is worth noting that human labor cost is not included in this manuscript, which makes our reported cost improvement lower.

### Whole genome sequencing ONT

Starting with 3–5 µg, samples were sheared using Covaris g-tubes (Covaris 520079) to achieve an average size of 15–20 kb. Sheared DNA was size selected on the PippinHT instrument (Sage Science) using the 6–10 kb High-Pass definition. Size-selected samples were used as input for the ONT SQK-LSK114 kit and libraries were prepared following the manufacturer’s instructions. Final libraries were eluted in 27 µL of the ONT elution buffer and quantified using the Qubit dsDNA quantification broad range assay (Thermo Fisher Scientific). Libraries were loaded at 15 fmoles and sequenced on R10.4.1 flowcells on the PromethION24 instrument.

### Whole genome sequencing PacBio

Starting with 5–10 µg of DNA, samples were sheared using g-tubes (Covaris 520079) to an average size of 15–20 kb. Sheared DNA was size-selected on the PippinHT instrument (Sage Science) using the 15–20 kb High-Pass definition. Size-selected samples were used as input for PacBio SMRTBell Prep Kit 3.0 for library preparation. DNA damage repair, A-tailing and adapter ligation were performed as per manufacturer’s instructions. SMRTBell Adapter Index Plate 96 A was used for barcoding each library. Adapter ligated DNA was nuclease treated following the manufacturer’s guidelines and purified using 1X PacBio SMRTBell Cleanup beads. Final libraries were quantified using the Qubit dsDNA quantification high-sensitivity assay (Thermo Fisher Scientific). Final library size was determined using the Agilent Femto Pulse. Each library was loaded at 325 pM on 1 Revio 25 M SMRT Cell and sequenced using SMRTLink v13 for a run time of 30 hours.

### Disease-related gene/BED file selection

Gene sets used for adaptive sampling were designed to be maximally inclusive while representing no more than 10% of the total base pairs of the genome. Each gene set thus comprises a comprehensive or near-comprehensive set of genes associated with the relevant phenotype or organ system. The neurodegeneration (*n* = 1730) gene set included all genes annotated in OMIM with the term ‘neurodegeneration’, as well as genes included in disease-specific gene panels from five independent commercially available diagnostic laboratories and the Genomics England PanelApp. The epilepsy (*n* = 1670) gene set included all genes included in at least two disease-specific gene panels from four independent commercially available diagnostic laboratories and the Genomics England PanelApp. The microcephaly (*n* = 1803) gene set included all genes annotated in OMIM^27^ with the term ‘microcephaly’, as well as genes included in disease-specific gene panels from five independent commercially available diagnostic laboratories and the Genomics England PanelApp^28^. The arthrogryposis (*n* = 2591) gene set included all genes annotated in OMIM with the term ‘arthrogryposis’, as well as genes included in disease-specific gene panels from four independent commercially available diagnostic laboratories and the Genomics England PanelApp. To further increase the size of the gene set, we additionally included genes indicated in the Human Protein Atlas to be highly expressed in muscle. The GENCODE v49 database was used to define gene coordinates, and BED files generated from each gene set inclusive of 10 kbp on either side of each gene.

### Basecalling and read alignment

Dorado v1.0.2 + c758d2f6^29^ basecalling and demultiplexing was first performed with command ‘dorado basecaller sup,5mCG_5hmCG {pod5} -r -x cuda:all --kit-name SQK-NBD114-96 -o {output_bam}’. The basecaller model was the latest at the time, i.e., ‘dna_r10.4.1_e8.2_400bps_sup@v5.2.0’. The output was further filtered based on the designed barcode in SQK-NBD114-96 kit. It is worth noting that we did not distinguish the accepted reads and rejected reads because even for rejected reads they are still barcoded to one of the samples in the trio, hence we could further perform the genome-wide variant calling and analysis even with rejected reads. Read alignment was performed after fastq file conversion with methylation MM, ML tag preserved. The reads are aligned to the reference hg38 no alt chromosome with minimap2^30^ v2.30-r1287 with ‘minimap2 -t 20 -a -Y -y -x map-ont -o {output.sam} {reference} {input.fastq}’ and further sorted and compressed in to bam file format. This workflow (Fig. 1a) results in 3 aligned bam files each for a barcode, which represents proband, mother, father, respectively.

### Sequencing coverage analysis

Mosdepth^31^ v0.3.11 was applied for sequencing coverage analysis for each barcode bam file with the information of target bed file. ‘mosdepth -t 10 -b {target.bed} -x {output} {input.bam}’ Sequencing depth within and outside of the targeted region was collected for sequencing quality assessment. HG002 trio-barcoded WGS coverage was obtained using the same command but without specifying a bed file.

### Variant calling, genotyping, and phasing

Small variant calling with Clair3^32^ v1.2.0 was ran for on each barcode bam files and each target regions with command ‘run_clair3.sh -b {input.bam} -f {reference} -m {model} --bed_fn = {target.bed} --sample_name = {barcode} -t 10 -p ont -o {output_folder}’. Small variants were phased with whatshap^33^ v2.8 ‘whatshap phase --reference {reference} -o {phased.vcf} {input.vcf} {input.bam}’ and reads were haplotagged with the resulting phased small variant callsets. Small variant callsets from the same family were then merged using bcftools^34^ v1.22. The variant count was obtained by the number of all SNVs discovered. Small variants counts were consolidated with ‘bcftools merge‘ and counted by ‘bcftools stats‘.

Structural variant discovery was performed with Sniffles2^35^ v2.6.3 on each barcode bam file with ‘sniffles -i {input.bam} --reference {reference} -v {output.vcf} --snf {output.snf} -t 10 --output-rnames’. We further intersected the SV callsets with targeted gene list regions bed file for the high-quality SV calls in the targeted regions. SVs which include rejected reads are called with ‘sniffles -i {input.bam} --reference {reference} -v {output.vcf.gz} --snf {output.snf} -t 10 --output-rnames --minsupport 2 --min-alignment-length 100’. The last two parameters were used to improve recall using the rejected reads which are typically short. SV counts were generated by ‘bcftools stats‘ on each barcode’s SV callsets. Structural variants from the probands were then jointly genotyped along with their parents’ SV callsets using kanpig^36^ v2.0.0-dev in trio mode.

Tandem repeats (TRs) were called over the adotto catalog TR regions^37^. TRs were called in ONT sequenced samples with medaka^38^ v2.1.0 and the command ‘medaka tandem --ignore_read_groups --model dna_r10.4.1_e8.2_400bps_sup@v5.2.0:consensus --phasing prephased {input.bam} {reference} {adotto_catalog} {sample_sex} {output}’. Three samples with tandem repeat expansions (*NAXE, DIP2B, FGF14* and *ATN1*) were later confirmed with PacBio replicates.

The variant calling in matched PacBio WGS samples was performed with PRINCESS^39^. TRs in PacBio replicates were called with TRGT^40^ v4.0.0 and the command ‘trgt genotype -g {reference} -r {bam} -b {adotto_catalog} -k {sample_sex} -o {output}’. TR results were consolidated and summarized using tdb and compared within families using custom scripts.

### HG002 benchmarking

Targeted CMRG region benchmarking: Small variants benchmarking was performed via rtg tools^41^ v3.13 vcfeval function. The small variant benchmarking catalog and bed region was obtained from GIAB ftp server. The ONT 30X sequenced data was obtained from ONT open data project and called with the same clair3 small variants and Sniffles SV calling command, and Illumina 30X sequenced data, small variants and SVs was obtained from a previous study^15^, in which we selected deepvariant^16^ v1.5 for small variants, Manta^42^ v1.6 for SVs since they performed the best in the dataset in that study.

TBAS Clair3 output inside the target CMRG region was compared against the benchmark’s baseline variant catalog with command ‘rtg vcfeval -b {CMRG.SNV.vcf} -c {input.vcf.gz} -e {CMRG.SNV.bed} -o {output} -t {reference}’. Genotype concordance was calculated among matched true-positive variants identified by RTG Tools vcfeval (records with INFO/BASE = “TP” and INFO/CALL = “TP”), thereby restricting evaluation to alleles successfully matched between truth and call sets. Concordance was defined as the fraction of non-missing diploid genotypes that were identical after converting phased to unphased representation and sorting alleles within each genotype to ensure phase- and order-invariant comparison. SV benchmarking was performed with truvari^43^ v5.3 and the command ‘truvari bench -c {input.vcf} -b {CMRG.SV.vcf} --includebed {CMRG.SV.bed} --refdist 1000 --reference {reference} -o {output}’ followed by ‘truvari refine --regions {output}/candidate.refine.bed --coords R --use-original-vcfs --align mafft --mafft-params ‘ --thread 4 ‘ {output}’. Phasing statistics in targeted regions were obtained by whatshap v2.8 with command ‘whatshap stats {input.vcf.gz}’.

For the whole-genome benchmarking including the rejected reads, the same procedures were followed with the benchmark’s baseline variant catalog from CMRG replaced with GIAB whole-genome catalog which is also accessible from the GIAB server.

DNA methylation benchmarking allows a maximum of ±0.2 methylation difference at each CpG position on GRCh38 and the CpG locations within this margin are counted as the same.

### Variant annotations

ANNOVAR^44^ (version 2025-03-02) was used on the family small variants VCF file. The following GRCh38 reference annotation databases were used:

- refGene (20211019) - all annotated transcripts in RefSeq gene
- hg38_avSNV151 (20170929) - dbSNV151
- hg38_dbnsfp47a (20240525) - predictive scores from dbNSFP(v3.0a)
- Clinvar_20250721 (20250721) - CLINVAR database with Variant Clinical Significance
- 1000g2015aug (20150824) − 1000 genome allele frequency
- ALL.sites.2015_08, AFR.sites.2015_08, AMR.sites.2015_08, EAS.sites.2015_08, EUR.sites.2015_08, SAS.sites.2015_08 − 1000 genome allele frequency separated by continental ancestry proportions clusters.
- Gnomad41_genome (20240602) - gnomAD genome collection (v4.1)

A tiering system was developed to filter variants based on their allele frequency, REVEL score, and CADD Phred. The system applies a minimum reporting threshold of allele frequency (AF) < 0.001 in the 1000 Genomes Project. To prioritize variants most likely to be causative, single-nucleotide variants (SNVs) were ranked by their REVEL and CADD scores. Tier 1 included homozygous and de novo SNVs with CADD > 20 or REVEL > 0.75, as well as those annotated with a ClinVar clinical significance (CLNSIG) label. Tier 2 comprised compound heterozygous SNVs meeting the same score thresholds. Tier 3 contained ultra-rare homozygous SNVs lacking ClinVar annotations, and Tier 4 included heterozygous SNVs meeting the same high-impact criteria as Tier 1. (Supplementary Data 4).

AnnotSV^45^ v3.5 was used to annotate the structural variants with ‘AnnotSV -SVinputFile {input.vcf}’pathogenetic candidate filtering was based on ACMG_class_norm field falling into ‘pathogenetic’ or ‘likely_pathogenetic’ categories.

STRchive^46^ was used for intersecting tandem repeat bed files to obtain the pathogenic repeats. Loci where the proband carries at least one tandem repeat allele distinct from both parents were identified and reported.

### Reporting summary

Further information on research design is available in the Nature Portfolio Reporting Summary linked to this article.

## Supplementary information

Supplementary Information

Description of Additional Supplementary Files

Supplementary Data 1-6

Reporting Summary

Transparent Peer Review file

## Source data

Source Data

## Data Availability

HG002 individual WGS sample was obtained from ONT public data project 2025.1 release https://epi2me.nanoporetech.com/giab-2025.01/. Illumina 30X deepvariant SNV and manta SV callsets were obtained from Behera et al.^15^. Benchmarking datasets: Genome wide SV: https://ftp-trace.ncbi.nlm.nih.gov/ReferenceSamples/giab/release/AshkenazimTrio/HG002_NA24385_son/NIST_SV_v0.6/ Genome wide SNV: https://ftp-trace.ncbi.nlm.nih.gov/ReferenceSamples/giab/release/AshkenazimTrio/HG002_NA24385_son/NISTv4.2.1/GRCh38/ CMRG regions: https://ftp-trace.ncbi.nlm.nih.gov/ReferenceSamples/giab/release/AshkenazimTrio/HG002_NA24385_son/CMRG_v1.00/ The sequencing data generated in this study have been deposited in the dbGap database under accession code phs003047.v3.p2. The sequencing data are available under restricted access for privacy reasons; access can be obtained by applying through dbGap. Source data are provided with this paper.

## Code availability

The codes and disease-related gene BED files are publicly available at https://github.com/Fu-Yilei/TBAS_pipeline

## References

[1] Nguengang Wakap, S. et al. Estimating cumulative point prevalence of rare diseases: analysis of the Orphanet database. *Eur. J. Hum. Genet.* **28**, 165–173 (2019). 10.1038/s41431-019-0508-0 PMC6974615 31527858

[2] Schmidt, E. M. et al. GREGOR: evaluating global enrichment of trait-associated variants in epigenomic features using a systematic, data-driven approach. *Bioinformatics* **31**, 2601 (2015). 10.1093/bioinformatics/btv201 PMC4612390 25886982

[3] Boycott, K. M. et al. A diagnosis for all rare genetic diseases: the horizon and the next frontiers. *Cell* **177**, 32–37 (2019). 10.1016/j.cell.2019.02.040 30901545

[4] Fu, Y., Timp, W. & Sedlazeck, F. J. Computational analysis of DNA methylation from long-read sequencing. *Nat. Rev. Genet.* **26**, 620–634 (2025). 10.1038/s41576-025-00822-5 PMC12882999 40155770

[5] Wojcik, M. H. et al. Beyond the exome: What’s next in diagnostic testing for Mendelian conditions. *Am. J. Hum. Genet* **110**, 1229–1248 (2023). 10.1016/j.ajhg.2023.06.009 PMC10432150 37541186

[6] Posey, J. E. et al. Insights into genetics, human biology and disease gleaned from family based genomic studies. *Genet. Med.* **21**, 798–812 (2019). 10.1038/s41436-018-0408-7 PMC6691975 30655598

[7] Miller, D. E. et al. Targeted long-read sequencing identifies missing disease-causing variation. *Am. J. Hum. Genet.* **108**, 1436–1449 (2021). 10.1016/j.ajhg.2021.06.006 PMC8387463 34216551

[8] Di Letto, P. et al. Nanopore sequencing solves an elusive case of sotos syndrome. *Am. J. Med. Genet. Part A* **197**, e64039 (2025). 10.1002/ajmg.a.64039 40062685

[9] Iyer, S. V., Goodwin, S. & McCombie, W. R. Leveraging the power of long reads for targeted sequencing. *Genome Res.* **34**, 1701–1718 (2024). 10.1101/gr.279168.124 PMC11610587 39567237

[10] Zheng, X. et al. STIX: Long-reads based accurate structural variation annotation at population scale. *bioRxiv* (2024) 10.1101/2024.09.30.615931.

[11] Rudaks, L. I. et al. Targeted long-read sequencing as a single assay improves the diagnosis of spastic-ataxia disorders. *Ann. Clin. Transl. Neurol.* **12**, 832–841 (2025). 10.1002/acn3.70008 PMC12040508 40007153

[12] Yeow, D. et al. Targeted long-read sequencing enables comprehensive analysis of the genetic and epigenetic landscape of inherited myopathies. *medRxiv* (2025) 10.64898/2025.12.06.25340828. PMC13470197 42401548

[13] Loose, M., Malla, S. & Stout, M. Real-time selective sequencing using nanopore technology. *Nat. Methods* **13**, 751 (2016). 10.1038/nmeth.3930 PMC5008457 27454285

[14] Wagner, J. et al. Curated variation benchmarks for challenging medically relevant autosomal genes. *Nat. Biotechnol.* **40**, 672–680 (2022). 10.1038/s41587-021-01158-1 PMC9117392 35132260

[15] Behera, S. et al. Comprehensive genome analysis and variant detection at scale using DRAGEN. *Nat. Biotechnol.* **43**, 1177–1191 (2024). 10.1038/s41587-024-02382-1 PMC12022141 39455800

[16] Poplin, R. et al. A universal SNP and small-indel variant caller using deep neural networks. *Nat. Biotechnol.* **36**, 983–987 (2018). 10.1038/nbt.4235 30247488

[17] Cetica, V. et al. Clinical and molecular characterization of patients with YWHAG-related epilepsy. *Epilepsia* **65**, 1439–1450 (2024). 10.1111/epi.17939 38491959

[18] Maroofian, R. et al. Biallelic MED27 variants lead to variable ponto-cerebello-lental degeneration with movement disorders. *Brain* **146**, 5031–5043 (2023). 10.1093/brain/awad257 PMC10690011 37517035

[19] Liu, Z. et al. Hemizygous variants in protein phosphatase 1 regulatory subunit 3F (PPP1R3F) are associated with a neurodevelopmental disorder characterized by developmental delay, intellectual disability and autistic features. *Hum. Mol. Genet.* **32**, 2981–2995 (2023). 10.1093/hmg/ddad124 PMC10549786 37531237

[20] Mitani, T. et al. High prevalence of multilocus pathogenic variation in neurodevelopmental disorders in the Turkish population. *Am. J. Hum. Genet.* **108**, 1981–2005 (2021). 10.1016/j.ajhg.2021.08.009 PMC8546040 34582790

[21] Ozaki, K. et al. Biallelic GGGCC repeat expansion leading to NAXE-related mitochondrial encephalopathy. *npj Genom. Med.* **9**, 1–12 (2024). 10.1038/s41525-024-00429-5 PMC11512015 39455596

[22] Pehlivan, D. et al. The genomics of arthrogryposis, a complex trait: candidate genes and further evidence for oligogenic inheritance. *Am. J. Hum. Genet.* **105**, 132 (2019). 10.1016/j.ajhg.2019.05.015 PMC6612529 31230720

[23] Pellerin, D. et al. Deep intronic FGF14 GAA repeat expansion in late-onset cerebellar ataxia. * N. Engl. J. Med.* **388**, e70 (2023). 10.1056/NEJMc2301605 37224216

[24] Eskandrani, A. et al. Recessive AFG3L2 Mutation Causes Progressive Microcephaly, Early Onset Seizures, Spasticity, and Basal Ganglia Involvement. *Pediatr. Neurol.* **71**, 24–28 (2017). 10.1016/j.pediatrneurol.2017.03.019 28449981

[25] Winnepenninckx, B. et al. CGG-repeat expansion in the DIP2B gene is associated with the fragile site FRA12A on chromosome 12q13.1. *Am. J. Hum. Genet.* **80**, 221–231 (2007). 10.1086/510800 PMC1785358 17236128

[26] Théberge, E. T. et al. Disco-interacting protein 2 homolog B CGG repeat expansion in siblings with neurodevelopmental disability and progressive movement disorder. *Mov. Disord.: Off. J. Mov. Disord. Soc.* **40**, 567–578 (2025). 10.1002/mds.30101 39854091

[27] Amberger, J. S., Bocchini, C. A., Scott, A. F. & Hamosh, A. Omim.org: leveraging knowledge across phenotype-gene relationships. *Nucleic Acids Res.* **47**, D1038–D1043 (2019). 10.1093/nar/gky1151 PMC6323937 30445645

[28] Martin, A. R. et al. PanelApp crowdsources expert knowledge to establish consensus diagnostic gene panels. *Nat. Genet.* **51**, 1560–1565 (2019). 10.1038/s41588-019-0528-2 31676867

[29] GitHub - nanoporetech/dorado: Oxford Nanopore’s Basecaller. *GitHub* https://github.com/nanoporetech/dorado.

[30] Li, H. Minimap2: pairwise alignment for nucleotide sequences. *Bioinformatics* **34**, 3094–3100 (2018). 10.1093/bioinformatics/bty191 PMC6137996 29750242

[31] Pedersen, B. S. & Quinlan, A. R. Mosdepth: quick coverage calculation for genomes and exomes. *Bioinformatics* **34**, 867 (2017). 10.1093/bioinformatics/btx699 PMC6030888 29096012

[32] Zheng, Z. et al. Symphonizing pileup and full-alignment for deep learning-based long-read variant calling. *Nat. Comput. Sci.* **2**, 797–803 (2022). 10.1038/s43588-022-00387-x 38177392

[33] Martin, M., Ebert, P. & Marschall, T. Read-based phasing and analysis of phased variants with WhatsHap. *Methods Mol. Biol.* **2590**, 127–138 (2023). 10.1007/978-1-0716-2819-5_8 36335496

[34] Danecek, P. et al. Twelve years of SAMtools and BCFtools. *GigaScience* **10**, giab008 (2021). 10.1093/gigascience/giab008 PMC7931819 33590861

[35] Smolka, M. et al. Detection of mosaic and population-level structural variants with Sniffles2. *Nat. Biotechnol.* **42**, 1571–1580 (2024). 10.1038/s41587-023-02024-y PMC11217151 38168980

[36] English, A. C., Cunial, F., Metcalf, G. A., Gibbs, R. A. & Sedlazeck, F. J. K-mer analysis of long-read alignment pileups for structural variant genotyping. *Nat. Commun.* **16**, 1–11 (2025). 10.1038/s41467-025-58577-w PMC11971316 40185777

[37] English, A. C. et al. Analysis and benchmarking of small and large genomic variants across tandem repeats. *Nat. Biotechnol.* **43**, 431–442 (2024). 10.1038/s41587-024-02225-z PMC11952744 38671154

[38] GitHub—nanoporetech/medaka: Sequence correction provided by ONT Research. *GitHub* https://github.com/nanoporetech/medaka.

[39] Mahmoud, M., Doddapaneni, H., Timp, W. & Sedlazeck, F. J. PRINCESS: comprehensive detection of haplotype resolved SNVs, SVs, and methylation. *Genome Biol.* **22**, 1–17 (2021). 10.1186/s13059-021-02486-w PMC8442460 34521442

[40] Dolzhenko, E. et al. Characterization and visualization of tandem repeats at genome scale. *Nat. Biotechnol.* **42**, 1606–1614 (2024). 10.1038/s41587-023-02057-3 PMC11921810 38168995

[41] Cleary, J. G. et al. Comparing Variant Call Files for performance benchmarking of next-generation sequencing variant calling pipelines. *bioRxiv* (2015) 10.1101/023754.

[42] Chen, X. et al. Manta: rapid detection of structural variants and indels for germline and cancer sequencing applications. *Bioinformatics* **32**, 1220–1222 (2016). 10.1093/bioinformatics/btv710 26647377

[43] English, A. C., Menon, V. K., Gibbs, R. A., Metcalf, G. A. & Sedlazeck, F. J. Truvari: refined structural variant comparison preserves allelic diversity. *Genome Biol.* **23**, 271 (2022). 10.1186/s13059-022-02840-6 PMC9793516 36575487

[44] Wang, K., Li, M. & Hakonarson, H. ANNOVAR: functional annotation of genetic variants from high-throughput sequencing data. *Nucleic Acids Res.* **38**, e164–e164 (2010). 10.1093/nar/gkq603 PMC2938201 20601685

[45] Geoffroy, V. et al. AnnotSV: an integrated tool for structural variations annotation. *Bioinformatics* **34**, 3572–3574 (2018). 10.1093/bioinformatics/bty304 29669011

[46] Hiatt, L. et al. STRchive: a dynamic resource detailing population-level and locus-specific insights at tandem repeat disease loci. *Genome Med.* **17**, 1–19 (2025). 10.1186/s13073-025-01454-4 PMC11938676 40140942

