# Supplementary material for: Cost-efficient long-read trio-barcoded adaptive sequencing improves rare disease diagnosis
Source: Nat Commun. 2026 Sep 4;17:10546. doi: 10.1038/s41467-026-77535-8 (PMC13642241; doi:10.1038/s41467-026-77535-8)
Supplement: Supplementary file 2 [file 41467_2026_77535_MOESM2_ESM.pdf]

Description of Additional Supplementary Files

Supplementary Data 1. Sequencing Summary of all ONT samples

Supplementary Data 2. Variant benchmarking on HG002 Trio

Supplementary Data 3. gene list for disease categories

Supplementary Data 4. All case details at the point of performing adaptive sampling

Supplementary Data 5. All pathogenetic SNV variant candidates. Tiers are described in methods

Supplementary Data 6. Tandem repeat expansion
