# Supplementary material for: Cost-efficient long-read trio-barcoded adaptive sequencing improves rare disease diagnosis
Source: Nat Commun. 2026 Sep 4;17:10546. doi: 10.1038/s41467-026-77535-8 (PMC13642241; doi:10.1038/s41467-026-77535-8)
Supplement: Supplementary file 4 [file 41467_2026_77535_MOESM5_ESM.pdf]

nature portfolio

Peer Review File

Cost-efficient long-read trio-barcoded adaptive sequencing improves rare disease diagnosis

Corresponding Author: Dr Fritz Sedlazeck

This manuscript has been previously reviewed at another journal. This document only contains information relating to versions considered at Nature Communications.

This file contains all reviewer reports in order by version, followed by all author rebuttals in order by version.

Version 0:

Reviewer comments:

Reviewer #1

(Remarks to the Author)
I thank the authors for responding to my comments. However, I do not believe the authors have sufficiently addressed the comments I have made. I note that my comments and views are shared by the other referees as well.

Starting with the fundamental question of novelty.

From the authors <<
However, we respectfully disagree that our work is similar in scope or intent. The two approaches differ fundamentally in both study design and application.

1. In Miller's work, targeted sequencing was applied to a single, pre-selected gene per individual, where the disease-causing structural variant had already been identified using prior clinical information and complementary sequencing technologies such as short-read sequencing, WES, or RNA-seq. In contrast, in our study, each proband is treated as an a priori unknown case, without prior knowledge of the causal locus.
2. Our approach leverages TBAS as a discovery-driven framework aimed at identifying pathogenic SVs, CNVs, and tandem repeats directly, rather than validating previously resolved variants. This distinction is critical: our method is designed to enable disease diagnosis, whereas Miller's approach primarily serves as a validation strategy. As a result, the scope, diagnostic utility, and cost-effectiveness of the two approaches are fundamentally different.
>>

Let's deal first with 1. In the work of Miller et al., the authors indeed have a priori evidence of a specific region to target. This is a pre-selected panel of a single gene. The authors of TBAS suggest that they have no prior knowledge of the causal locus and that each probing is treated as an a priori unknown case. In this case, the authors start with a panel of not one gene, but 395 genes based on the CMRG regions (line 53). In figure 1 panel A, they demonstrate the use of a priori information (based on phenotype inspection) to generate some panels based on different presentations. Subsequently, the panels for patients are derived from OMIM (lines 113-115). This is directly analogous to the work of Miller et al. and also any other adaptive sampling paper that predicts a panel of target genes based on phenotype. The only difference is the number of genes in the panel.

With respect to 2, I do not see the difference with any prior application of trio-based sequence analysis or adaptive sampling method. The two approaches are not fundamentally different. The panel is identified based on pre-existing information, and sequencing occurs with downstream subsequent analysis. There is nothing inherent to the sequencing process that discovers the variant of significance.

My view is fundamentally unchanged.

I also note that my comment is exactly that made by reviewer 2. In essence, this manuscript is the combination of prior existing approaches in an obvious way.

The authors are correct that the readfish paper I cited utilised cell lines. But again, there is little novelty in showing that this works from patient samples. The ability to analyse SNVs and methylation in patient samples was previously demonstrated in numerous adaptive sampling papers.

I fundamentally disagree with the authors' assertion that they have developed a novel framework and approach. It is interesting to note that the authors acknowledge the use of a priori information in developing the panel (an initial OMIN <sic> classification of phenotypes). I refer to this repeatedly in my comments below.

TBAS (Trio Barcoded ONT Adaptive Sampling) is simply the selection of a panel of genes based on phenotype. That panel is then applied to three samples run barcoded on the same flow cell. Subsequently, analysis of the trio data reveals that panel-based diagnoses can be made. Interestingly, some variants are missed from the panel but can be recovered.

The authors state that the HG002 analysis provides a benchmark for the disease cases in the context of WGS versus adaptive sampling, but this simply is not true. The critical question with the use of any adaptive sampling approach is if, in patients, it missed anything of importance. To be shown to have utility for clinical application, that comparison is essential. The manuscript goes on to identify more than one case where this occurred.

In response to my comment on "what is 'near-complete' variant phasing," the authors respond:
<<
We thank the reviewer for pointing out the ambiguity. The term "near-complete" variant phasing is indeed misleading, and we corrected this (see below sentence). Indeed, we have two ways to phase the variants. First, using long-reads only. Here, we are able to phase variants in the targeted regions, including novel variants, as demonstrated in our paper (CASE BH14008). For HG002, we achieve 99.5% of variants phased in the targeted regions.

Second, since we have the trios sequenced, we also can leverage that to do trio phasing. This phases all variants (except for the de novo variants).

"TBAS achieved a high variant phasing rate and detection of small variants, structural variants, and tandem repeats with high accuracy and a high potential solve rate."
>>

I do not exactly understand the distinction the authors are making here. When they say "long-reads only" do they mean just the targeted reads? Or long reads from the proband only? Then when they subsequently state that they "have the trios sequenced" do they mean they use the data from the trio sequenced using the TBAS approach or are they utilising other independent datasets for that sample? Again - the line "TBAS achieved" is misleading (see comments below).

In the comment with respect to line 21 the authors state: <<
"However, we are unable to share the detailed breakdown of the cost in the manuscript since people can back-calculate by subtracting the reagent costs, which is harmful to our sequencing centre."
>>
The local costs can of course be hidden, but the major costs are the commercial costs of the flowcells and reagents - the authors should simply quote the current list prices and explain how cost savings are made.

With respect to my original question 17. My comment was that SV calling should be enhanced compared to Illumina data (which it is) but that the comparator ONT data (non-adaptive run on one flowcell) would be extremely unlikely to meet the depth required for a satisfactory analysis.

Detailed specific comments:

I note that the authors addressed my illustrative comment (comment 20) - but this same issue arises multiple other times in the manuscript. I have highlighted all the ones I have seen below. Many of them I had previously commented on.

Statements such as that on line 125 "TBAS targeted 1,704 genes" are wholly misleading. This should state something along the lines of "Based on the patient phenotype, 1,704 genes were selected to help resolve 5 examples of a neurodegenerative phenotype". There was no algorithmic automated analysis.

Similarly, the subsequent sentence "TBAS was able to independently discover the causative variants…". No. The causative variant was within the pre-selected panel of 1,704 genes selected for targeting. Subsequent standard analysis pipelines released the underlying causative variant. Line 128 "TBAS successfully identified the CLP1".

The way the manuscript is presented suggests that this approach autonomously can identify the causative variants. But this simply is not true. Adaptive sampling allows the easy development of a panel of target genes based on phenotype. Subsequent analysis of those resultant data allows a clinical geneticist to assign a likely causative agent. In the absence of whole genome long read sequencing for these trios, the authors cannot demonstrate that they have not missed additional key variants. Indeed, this flaw is present in their own data. Line 133 TBAS is presented as being able to recover this variant via rejected read calling - OK this is a good example of salvage but it is unclear to me if the TBAS pipeline shows this, or the authors had to use a priori knowledge from the previous exome analysis to go in and analyse the data to specifically recover that variant. As the authors now acknowledge on lines 135-136, it would have made sense to utilise the benefit of adaptive

sampling to just add that candidate into the experiment.

Line 140-142, the authors discuss the detection of an expansion that was not detected by Illumina sequencing. They ascribe this to “the improved resolution of the TBAS approach”. No - this has nothing to do with the TBAS approach, rather it is the use of long reads instead of short reads. This variant would have been resolved by long read WGS on Nanopore, PacBIO, or even, most likely, newer Illumina methods for artificial long reads. This is not a specific property of TBAS. I note this was the point I was making in my previous comment 24. The authors need to be clear about where the benefit is derived from. It is not a property of running TBAS - it is a property of using long rather than short reads.

Line 146. “The diagnostic power of TBAS” - again, no. This is not a property of TBAS, but a property of long read sequencing in conjunction with existing trio analysis methods.

Line 149: “TBAS targeted 2,582 genes” - again, no. TBAS did not target anything. A panel appropriate to the phenotype was chosen by the authors.

Lines 152-158, I’m unclear on the point the authors are making here. The use of the adaptive sampling panel identifies the CHRNG mutation - is this sufficient explanation? Does the use of Trio analysis not help resolve this in any way?

Line 158: “TBAS found a homozygous frameshift variant” - this is the same sort of wording that is misleading. Analysis of the sequence data revealed this - but again, this would be found by any other long read sequencing method.

Line 163 - the wording here is better - perhaps “where the TBAS approach was used to” would be better? Line 172 is also useful in this regard - the fundamental underlying improvement here is the use of long read sequencing with trios. The application of adaptive sampling, when a panel is sensibly chosen, does not limit the ability to detect. Unfortunately, we are back at Line 177 to the “TBAS independently discovered”.

Line 181/182 - no genes were targeted by TBAS. The authors selected a panel based on the phenotype. Unfortunately, lines 193-195 highlight again the potential issues with using a panel in these contexts, and the fact that these can be resolved is of use.

Line 212. For clarification, is there any new algorithmic or analytical approach that did not exist prior to this paper in the TBAS pipeline? From my studying of the pipeline, I do not believe this to be the case. If there is something novel, the authors should clarify this. I note that the authors suggest this in response to point 3 from reviewer 2. I do not believe they have clarified that point at all. Furthermore, they have completely overstated in the rebuttal that “there is no method that can process raw ONT data into clinical variants for a trio”. How about ONT’s own wf-trio?

Line 218 - where is the evidence for this? In my experience, small panels can work very efficiently. Similarly, lines 219-221 - where is the evidence to support this? I could not find any data presented in the methods. This is reflected in comments from the authors in response to my question (9, 10, 13, etc.). It’s worth noting that the authors state in response to question 13 that small panels limit enrichment efficiency, yet in answer to question 10, state that the increased coverage seen is a consequence of the smaller panel used.

(Remarks on code availability)
The code is now described as a pipeline which is more accurate and no longer requires a slurm cluster.

The code recommends a minimum (?) python version of 3.9 - its worth noting that python 3.9 is end of life and no longer receiving security or maintenance updates. If the authros can bump the minimum python version up it will enable better uptake. Similarly, stating:

"Standard long-read analysis command-line tools used by the pipeline: samtools, minimap2, sniffles, mosdepth, run_clair3.sh, kanpig, bedtools, bgzip, bcftools, whatshap, medaka, modkit, tdb."

This should really be a conda yml file or similar - i.e make it simple for users to adopt. Currently this requires a level of bioinformatics ability that is not commonly available.

I stopped testing the code at this point.

I was hoping there would be some explanation in the README or elsewhere in the repository of the novelty in the analysis and the underlying assummptions/approach but there is no reference to anythign other than chaining of existing tools.

Reviewer #3

(Remarks to the Author)
I thank the authors for the effort they have made to improve the manuscript.

I prefer the new title and I am happy with how the variant calling / phasing benchmarks are now presented, and how performance in on-target vs off-target regions is reported.

I feel there is still room for improved clarity in the section on the 13 x trios with rare disease:

1. Please add a section to the Methods to describe the process and criteria by which the 13 cases were selected; what sequencing and or other testing methods had been performed previously; and the extent of previous data analysis. You've provided some of this information in the reviewer rebuttal, but it needs to be added to the main manuscript. Please clarify that the TBAS team/analysts were blind to and previous diagnoses. If selection of cases by the referring clinicans was purely random, please state this.

2. For the 5/8 trio cases that were newly solved using TBAS, there are several instances where good candidate variants identified by TBAS "were later confirmed on reassassement of exome data". Please clarify why those variants were not detected/prioritised during previous analysis. I think it is valuable and important to distinguish these kind of cases from those where TBAS found a genuinely challenging causative variant that could not be detected with short read data. From what I can gather, all cases apart from the NAXE and possibly the ATN1 repeat expansions, were solvable with existing short-read data. Maybe I'm misinterpreting. Please clarify.

If these points can be addressed then I would be supportive of publication.

I'd also point the reviewers to two similar studies I was involved with, where we used barcoded ONT adaptive sampling to improve rare disease diagnosis (2-4 patients per flow cell, though not specifically focused on family trios like TBAS), which might optionally be cited. DOIs:
10.1002/acn3.70008
10.64898/2025.12.06.25340828

All the best

Ira Deveson

(Remarks on code availability)
I concur with Reviewer 1's comments that the code here should not be described as novel tool or software package. The code provided is appropriate to document/reproduce the method followed for this study, but it is not a generic, easily deployable package that others could easily adopt, especially in a clinical context.

Open Access This Peer Review File is licensed under a Creative Commons Attribution 4.0 International License, which permits use, sharing, adaptation, distribution and reproduction in any medium or format, as long as you give appropriate credit to the original author(s) and the source, provide a link to the Creative Commons license, and indicate if changes were made.
In cases where reviewers are anonymous, credit should be given to 'Anonymous Referee' and the source.
The images or other third party material in this Peer Review File are included in the article's Creative Commons license, unless indicated otherwise in a credit line to the material. If material is not included in the article's Creative Commons license and your intended use is not permitted by statutory regulation or exceeds the permitted use, you will need to obtain permission directly from the copyright holder.
To view a copy of this license, visit https://creativecommons.org/licenses/by/4.0/

We thank the reviewers for their continued evaluation of our manuscript. Their points helped to improve the clarity of the manuscript and to highlight the essential points across the method. We have now changed several formulations of TBAS in the main text thanks to Reviewer 1 comments and also have integrated more details about the knowledge of the patients at the time we conducted the experiments. Below we answered each of the reviewers concerns and questions and tried ot clarify certain points. Like before our replies are in blue. We also kept track changes of the current modifications for the main text in blue as well.

Reviewer #1 (Remarks to the Author)

I thank the authors for responding to my comments. However, I do not believe the authors have sufficiently addressed the comments I have made. I note that my comments and views are shared by the other referees as well.

Starting with the fundamental question of novelty.

From the authors <<

However, we respectfully disagree that our work is similar in scope or intent. The two approaches differ fundamentally in both study design and application.

1. In Miller's work, targeted sequencing was applied to a single, pre-selected gene per individual, where the disease-causing structural variant had already been identified using prior clinical information and complementary sequencing technologies such as short-read sequencing, WES, or RNA-seq. In contrast, in our study, each proband is treated as an a priori unknown case, without prior knowledge of the causal locus.

2. Our approach leverages TBAS as a discovery-driven framework aimed at identifying pathogenic SVs, CNVs, and tandem repeats directly, rather than validating previously resolved variants. This distinction is critical: our method is designed to enable disease diagnosis, whereas Miller's approach primarily serves as a validation strategy. As a result, the scope, diagnostic utility, and cost-effectiveness of the two approaches are fundamentally different.

>>

Let's deal first with 1. In the work of Miller et al., the authors indeed have a priori evidence of a specific region to target. This is a pre-selected panel of a single gene. The authors of TBAS suggest that they have no prior knowledge of the causal locus and that each probing is treated as an a priori unknown case. In this case, the authors start with a panel of not one gene, but 395 genes based on the CMRG regions (line 53). In figure 1 panel A, they demonstrate the use of a priori information (based on phenotype inspection) to generate some panels based on different presentations. Subsequently, the panels for patients are derived from OMIM (lines 113-115). This is directly analogous to the work of Miller et al. and also any other adaptive sampling paper that predicts a panel of target genes based on phenotype. The only difference is the number of genes in the panel.

We thank the reviewer for this clarification. We agree that our previous wording was too strong if it implied that TBAS is performed without any a priori biological information. TBAS is phenotype guided, and the adaptive sampling targets are selected before sequencing based on the clinical presentation and disease-associated gene resources such as OMIM.

The distinction we intended to make is not "no prior information" versus "prior information," but rather "known candidate-locus follow-up" versus "phenotype-guided analysis of unresolved trios." In the Miller et al. study, adaptive sampling was applied to a specific pre-selected gene or locus for which prior clinical and molecular evidence already implicated the one specific region. In TBAS, the rare-disease trios were unresolved at the

time of analysis, and the disease-causing variant or locus was not known to the TBAS analysis team. The adaptive sampling panel was therefore not designed around a known candidate variant, but around broad phenotype categories. These panels included thousands of genes and covered approximately 6 to 8% of the genome, requiring balanced trio barcoding, uniform enrichment across a much larger target space, and downstream inheritance-aware analysis across SNVs, SVs, tandem repeats and methylation.

The CMRG experiment in HG002 was not intended as a rare-disease discovery experiment. It was a technical benchmark using well-characterized challenging medically relevant genes to evaluate coverage, variant calling, phasing and methylation performance. The rare-disease application was then tested in 13 unresolved GREGoR trios using phenotype-selected panels.

Thus, while both approaches use adaptive sampling and prior biological knowledge to define targets, TBAS is not simply a larger single-gene validation experiment such as the study from Miller et al. It demonstrates a cost-reduced, phenotype-guided long-read trio workflow for unresolved rare-disease cases in which the candidate variant is not known before sequencing.

With respect to 2, I do not see the difference with any prior application of trio-based sequence analysis or adaptive sampling method. The two approaches are not fundamentally different. The panel is identified based on pre-existing information, and sequencing occurs with downstream subsequent analysis. There is nothing inherent to the sequencing process that discovers the variant of significance.

We have to respectfully disagree but maybe let us try to make it clearer. TBAS changes the sequencing experiment used for rare-disease trio analysis. By combining trio barcoding with phenotype-informed adaptive sampling, TBAS concentrates long-read sequencing capacity on clinically relevant genomic regions while preserving the ability to detect SNVs, SVs, tandem repeat expansions, haplotypes, and methylation from native molecules. This distinguishes TBAS from conventional trio sequencing, where all three genomes are sequenced separately or at lower effective target coverage, and from prior adaptive sampling applications that didn't include the phenotype component nor establish a complete trio-based rare-disease diagnostic workflow. The result is a scalable strategy for phenotype-guided long-read trio sequencing that reduces cost while retaining all the variant classes and molecular context most relevant to unresolved rare disease.

My view is fundamentally unchanged.

I also note that my comment is exactly that made by reviewer 2. In essence, this manuscript is the combination of prior existing approaches in an obvious way.

The authors are correct that the readfish paper I cited utilised cell lines. But again, there is little novelty in showing that this works from patient samples. The ability to analyse SNVs and methylation in patient samples was previously demonstrated in numerous adaptive sampling papers.

This distinction is not simply an application to patient samples. Rare-disease trio sequencing requires simultaneous interpretation of three related genomes, balanced enrichment across barcodes, inheritance-aware variant calling and sufficient coverage for heterogeneous variant classes. These requirements are not addressed by demonstrating adaptive sampling in cell lines or by showing that SNVs and methylation can be analyzed from patient samples. In our study, TBAS achieved high coverage across phenotype-selected disease regions, benchmarked small variants and structural variants against GIAB CMRG regions, preserved phasing and methylation information, and recovered known or candidate diagnostic variants across SNVs, tandem repeats and methylation-linked repeat expansions. Thus, the contribution is a validated, cost-reduced sequencing and analysis framework for long-read rare-disease trios, rather than a claim that adaptive sampling or trio analysis alone is novel.

I fundamentally disagree with the authors' assertion that they have developed a novel framework and approach. It is interesting to note that the authors acknowledge the use of a priori information in developing the panel (an initial OMIN <sic> classification of phenotypes). I refer to this repeatedly in my comments below.

We do not claim that adaptive sampling, trio interpretation, methylation calling, or SNV/SV/TR analysis are individually new. The contribution of TBAS is the implementation and benchmarking of these components as a single rare-disease trio sequencing strategy/worflow. This includes phenotype-informed target selection, proband-parent barcoding on one flow cell, adaptive enrichment, long-read detection of multiple variant classes, read-backed phasing, inheritance-aware interpretation, methylation analysis and clinical prioritization.

TBAS (Trio Barcoded ONT Adaptive Sampling) is simply the selection of a panel of genes based on phenotype. That panel is then applied to three samples run barcoded on the same flow cell. Subsequently, analysis of the trio data reveals that panel-based diagnoses can be made. Interestingly, some variants are missed from the panel but can be recovered.

The authors state that the HG002 analysis provides a benchmark for the disease cases in the context of WGS versus adaptive sampling, but this simply is not true. The critical question with the use of any adaptive sampling approach is if, in patients, it missed anything of importance. To be shown to have utility for clinical application, that comparison is essential. The manuscript goes on to identify more than one case where this occurred.

We agree that the HG002 CMRG experiment is a technical benchmark rather than a complete demonstration that no clinically relevant variant would be missed in patient samples. This was never stated to be otherwise in our manuscript. The HG002 experiment benchmarks on-target variant calling, phasing and methylation performance in a well-characterized truth-set region. It does not replace genome-wide long-read sequencing and cannot prove that phenotype-selected adaptive sampling captures every relevant variant in an unresolved patient.

To resolve this we now added "This experiment was used as a technical benchmark of coverage, variant calling, phasing and methylation performance, not as a rare-disease discovery experiment."

In response to my comment on "what is 'near-complete' variant phasing," the authors respond:

<<

We thank the reviewer for pointing out the ambiguity. The term "near-complete" variant phasing is indeed misleading, and we corrected this (see below sentence). Indeed, we have two ways to phase the variants. First, using long-reads only. Here, we are able to phase variants in the targeted regions, including novel variants, as demonstrated in our paper (CASE BH14008). For HG002, we achieve 99.5% of variants phased in the targeted regions.

Second, since we have the trios sequenced, we also can leverage that to do trio phasing. This phases all variants (except for the de novo variants).

"TBAS achieved a high variant phasing rate and detection of small variants, structural variants, and tandem repeats with high accuracy and a high potential solve rate."

>>

I do not exactly understand the distinction the authors are making here. When they say “long-reads only” do they mean just the targeted reads? Or long reads from the proband only? Then when they subsequently state that they “have the trios sequenced” do they mean they use the data from the trio sequenced using the TBAS approach or are they utilising other independent datasets for that sample? Again - the line “TBAS achieved” is misleading (see comments below).

We apologize for the confusion. The reviewer is correct that our previous reply did not clearly distinguish the two phasing analyses.

By “long-read only phasing,” we meant read-backed phasing using the TBAS long reads from a single individual barcode, without using parental genotypes or any external sequencing dataset. In practice, this corresponds to phasing heterozygous variants within the targeted regions using the long-read alignments from the proband, mother or father separately. In the HG002 benchmark, this read-backed analysis phased 99.5% of variants in the targeted regions. This approach can sometimes assign de novo variants to a haplotype when they are linked by long reads to nearby phased heterozygous variants

Separately, because TBAS sequences the proband and both parents on the same barcoded flow cell, the same TBAS dataset can also be used for trio-based inheritance analysis. Here, we use the TBAS-derived genotypes from all three family members to determine whether variants are inherited, de novo, homozygous, or compound heterozygous where parental genotypes are informative. This analysis does not use independent datasets. De novo variants cannot be phased by Mendelian transmission alone, although they can sometimes be assigned to a proband haplotype if the variant is linked by long reads to nearby phased heterozygous variants.

We therefore now distinguish between read-backed phasing within targeted regions and inheritance-aware trio interpretation from the same TBAS run: ”Furthermore, TBAS achieved long-read based phasing for 99.5% of all variants.”

In the comment with respect to line 21 the authors state: <<

“However, we are unable to share the detailed breakdown of the cost in the manuscript since people can back-calculate by subtracting the reagent costs, which is harmful to our sequencing centre.”

>>

The local costs can of course be hidden, but the major costs are the commercial costs of the flowcells and reagents - the authors should simply quote the current list prices and explain how cost savings are made.

We agree and have revised the cost analysis using publicly available ONT list prices:

<u>TBAS trio on one PromethION flow cell</u>

- 1 PromethION flow cell: $1,040
- 1 Native Barcoding Kit reaction for the trio: $79.17
- Total ONT sequencing consumables: $1,119.17 per trio

<u>Conventional ONT long-read WGS trio as three separate single-sample runs</u>

- 3 PromethION flow cells: 3 × $1,040 = $3,120
- 3 Ligation Sequencing Kit reactions: 3 × $120 = $360
- Total ONT sequencing consumables: $3,480 per trio

TBAS consumable cost = $1,119.17 / $3,480 = 32.2% of the conventional three-flow-cell ONT trio WGS strategy. Our initial reported cost improvement was lower due to inclusion of personal costs etc.

We thus changed the main text to “Using public ONT list prices, TBAS reduces estimated ONT sequencing consumable costs to 32.2% of a conventional three-flow-cell ONT long-read trio WGS strategy,”

This has now been reported in the methods section in detail.

With respect to my original question 17. My comment was that SV calling should be enhanced compared to Illumina data (which it is) but that the comparator ONT data (non-adaptive run on one flowcell) would be extremely unlikely to meet the depth required for a satisfactory analysis.

We thank the reviewer for pointing this out. This is exactly our point in the paper that if we are using TBAS instead of trio-barcoded ONT whole-genome sequencing we can improve the coverage depth for the better quality variant analysis and phasing. In fact, Sniffles2 and clair3 can work on 12x long-read but with a relatively lower coverage, and in our manuscript we showcased the improvement in the targeted regions from TBAS given the higher coverage per barcode.

“The CMRG benchmark regions measured TBAS performance with F1-scores of 94.95% for small variants and 92.19% for SV (Supplementary Table 2), exceeding the performance of TBWGS at 91.74% and 89.95%, respectively.”

Detailed specific comments:

I note that the authors addressed my illustrative comment (comment 20) - but this same issue arises multiple other times in the manuscript. I have highlighted all the ones I have seen below. Many of them I had previously commented on.

Statements such as that on line 125 “TBAS targeted 1,704 genes” are wholly misleading. This should state something along the lines of “Based on the patient phenotype, 1,704 genes were selected to help resolve 5 examples of a neurodegenerative phenotype”. There was no algorithmic automated analysis.

We thank the review for pointing this out, we have corrected the similar statement in multiple locations in our manuscript:

“TBAS targeted 1,704 genes” -> “Based on the patient phenotype, 1,704 genes were selected to help resolve 5 examples of neurodegenerative phenotype class trios.”

Similarly, the subsequent sentence “TBAS was able to independently discover the causative variants…”. No. The causative variant was within the pre-selected panel of 1,704 genes selected for targeting. Subsequent standard analysis pipelines released the underlying causative variant. Line 128 “TBAS successfully identified the CLP1”.

“TBAS was able to independently discover the causative variants…” -> “Analysis of the TBAS data prioritized the known causative variants in all previously solved cases and identified one strong candidate in the three previously unsolved cases”

“TBAS successfully identified the CLP1”. -> “the pipeline correctly ranked CLP1 (NM_006831.3):c.419G>A, p.Arg140His homozygous variant in two previously solved cases (Supplementary 2.1.1) as the highest rank causative variants”

The way the manuscript is presented suggests that this approach autonomously can identify the causative variants. But this simply is not true. Adaptive sampling allows the easy development of a panel of target genes based on phenotype. Subsequent analysis of those resultant data allows a clinical geneticist to assign a likely causative agent. In the absence of whole genome long read sequencing for these trios, the authors cannot demonstrate that they have not missed additional key variants. Indeed, this flaw is present in their own data. Line 133 TBAS is presented as being able to recover this variant via rejected read calling - OK this is a good example of salvage but it is unclear to me if the TBAS pipeline shows this, or the authors had to use a priori knowledge from the previous exome analysis to go in and analyse the data to specifically recover that variant. As the authors now acknowledge on lines 135-136, it would have made sense to utilise the benefit of adaptive sampling to just add that candidate into the experiment.

We thank the reviewer for making the insightful comment. We had to use the prior exome sequencing knowledge to retrieve this SNP, and now we made this clear in the paper as “TBAS recovered this variant based on a priori knowledge from the paired exome analysis”. Thus as we mentioned in our response last time this was never counted as the solved case. Nevertheless, it still shows TBAS is capable of recovering the DNA variant and we do not need to sequence again.

Line 140-142, the authors discuss the detection of an expansion that was not detected by Illumina sequencing. They ascribe this to “the improved resolution of the TBAS approach”. No - this has nothing to do with the TBAS approach, rather it is the use of long reads instead of short reads. This variant would have been resolved by long read WGS on Nanopore, PacBIO, or even, most likely, newer Illumina methods for artificial long reads. This is not a specific property of TBAS. I note this was the point I was making in my previous comment 24. The authors need to be clear about where the benefit is derived from. It is not a property of running TBAS - it is a property of using long rather than short reads.

We thank the review of pointing this out, we have changed the sentence into “highlighting the value of the long-read component of TBAS for resolving repeat expansions that are difficult to assess with short-read sequencing”

Line 146. “The diagnostic power of TBAS” - again, no. This is not a property of TBAS, but a property of long read sequencing in conjunction with existing trio analysis methods.

We thank the reviewer for mentioning this, we have removed the “diagnostic power” and changed the sentence into “highlight the capability of lon reads generated from the TBAS approach in resolving pathogenic repeat expansions”

Line 149: “TBAS targeted 2,582 genes” - again, no. TBAS did not target anything. A panel appropriate to the phenotype was chosen by the authors

“TBAS targeted 2,582 genes” -> “For the arthrogryposis phenotype class, phenotype group, 2,582 genes were selected and TBAS paired with the bioinformatic approach identified an average of 314,131 SNV and 3,041 SV across trios.”

Lines 152-158, I’m unclear on the point the authors are making here. The use of the adaptive sampling panel identifies the CHRNG mutation - is this sufficient explanation? Does the use of Trio analysis not help resolve this in any way?

The trio analysis helped us to identify this variant as in the parents are both heterozygous and the proband is homozygous, which we now mention in the supplementary information “TBAS identified two homozygous missense variants of potential clinical relevance while both parents are carrying the heterozygous variants.”.

Line 158: “TBAS found a homozygous frameshift variant” - this is the same sort of wording that is misleading. Analysis of the sequence data revealed this - but again, this would be found by any other long read sequencing method.

We have changed “TBAS found a homozygous frameshift variant” to “The TBAS analysis pipeline ranked SNVs as the top candidates in two solved cases.”

Line 163 - the wording here is better - perhaps “where the TBAS approach was used to” would be better? Line 172 is also useful in this regard - the fundamental underlying improvement here is the use of long read sequencing with trios. The application of adaptive sampling, when a panel is sensibly chosen, does not limit the ability to detect. Unfortunately, we are back at Line 177 to the “TBAS independently discovered”.

We have changed “TBAS independently discovered” into “The paired bioinformatic pipeline based on TBAS data discovered SNV as the top candidates of two solved cases”

Line 181/182 - no genes were targeted by TBAS. The authors selected a panel based on the phenotype. Unfortunately, lines 193-195 highlight again the potential issues with using a panel in these contexts, and the fact that these can be resolved is of use.

We thank the reviewer for pointing this out but we changed the words into “Lastly, we further investigated a solved and two unsolved trios associated with microcephaly and 1,769 genes selected based on the phenotype and used as the adaptive sampling target panel of the TBAS workflow" because we think the selected gene can be targeted by TBAS because adaptive sampling is a targeted approach.

Line 212. For clarification, is there any new algorithmic or analytical approach that did not exist prior to this paper in the TBAS pipeline? From my studying of the pipeline, I do not believe this to be the case. If there is something novel, the authors should clarify this. I note that the authors suggest this in response to point 3 from reviewer 2. I do not believe they have clarified that point at all. Furthermore, they have completely overstated in the rebuttal that “there is no method that can process raw ONT data into clinical variants for a trio”. How about ONT’s own wf-trio?

We agree and reported that the pipeline uses established tools for alignment, variant calling, phasing, methylation analysis and annotation. Its contribution is the integration of these tools into a reproducible TBAS-specific workflow that starts from barcoded ONT data and produces ranked candidate variants for rare-disease trio review.

The analytical approach of the bioinformatic pipeline has 1. An automatic ranking based on the anovar output and variant calling result. 2. Novelty of combining methylation and variant calling approach in an automated trio fashion. 3. Especially for the tandem repeat part, no pipeline has before compared the length among trios so we know the proband has an elevated repeat length compared to the parents. We would say the bioinformatic approach paired with TBAS has novelty especially in incorporating the strength of long-read sequencing.

wf-trio has no variant annotations step which makes the workflow merely a variant calling approach. However, the outcome from our pipeline was directly used by the clinicians so they can investigate the highly ranked variants and see their potential of being the causative variants.

Line 218 - where is the evidence for this? In my experience, small panels can work very efficiently. Similarly, lines 219-221 - where is the evidence to support this? I could not find any data presented in the methods. This is reflected in comments from the authors in response to my question (9, 10, 13, etc.). It's worth noting that the authors state in response to question 13 that small panels limit enrichment efficiency, yet in answer to question 10, state that the increased coverage seen is a consequence of the smaller panel used.

By not being efficient here we mean the increased coverage does not scale linearly with the reduced panel size. We only reached less than 2 fold coverage increase by reducing the panel size by 10 fold.

We have now revised the closing paragraph: "The TBAS workflow provides a cost-efficient and comprehensive strategy for phenotype-guided long-read trio analysis in rare disease. Across 13 GREGoR trios, TBAS recovered the known candidate variant in all five previously solved cases and prioritized likely causative or clinically relevant candidates in five of eight previously unresolved cases, resulting in a 76.9% potential solve rate. Across the previously unresolved cases, TBAS contributed in two complementary ways. For repeat expansion cases, especially *NAXE* and *ATN1*, the long-read data enabled direct allele sizing, inheritance assessment and, in the *NAXE* case, methylation support around the expanded allele. In other cases, the TBAS workflow highlighted and reported clinically relevant SNV or indel candidates that had not been prioritized during earlier clinical review. Retrospective inspection of prior short-read or exome data showed that some of these variants were technically present, but had not been recognized as leading candidates before TBAS analysis. Thus, the added value of TBAS was not limited to detecting variants absent from short-read data. It also provided independent trio-based prioritization and molecular interpretation across variant classes. The accompanying new variant calling and annotation pipeline includes variant prioritizations to enable wide scale usage of this approach. Furthermore, the increased coverage of TBAS would allow for the detection of mosaic variants, although we couldn't demonstrate this in our cases. Unlike hybrid capture, adaptive sequencing still provides partial access to off-target regions, offering additional contextual information, though at lower coverage and reduced variant-calling sensitivity. However, very small target panels (e.g., 0.6% in HG002) limit enrichment efficiency, and could lead to imbalance barcode coverage, illustrating that adaptive sequencing is most effective when applied to moderately sized, phenotype-informed gene sets. In practice, we have found out the optimal shearing of DNA is 10-12 kbp long and the optimal reference genome span is 5-10% (**methods**). Together, these results establish TBAS as a scalable, cost-efficient, phenotype-guided long-read sequencing framework for rare-disease trio analysis."

(Remarks on code availability)

The code is now described as a pipeline which is more accurate and no longer requires a slurm cluster.

The code recommends a minimum (?) python version of 3.9 - its worth noting that python 3.9 is end of life and no longer receiving security or maintenance updates. If the authros can bump the minimum python version up it will enable better uptake. Similarly, stating:

We thank the reviewer for noticing this, the version is indeed the minimum required, and we have now bumped the version to 3.10.

"Standard long-read analysis command-line tools used by the pipeline: samtools, minimap2, sniffles, mosdepth, run_clair3.sh, kanpig, bedtools, bgzip, bcftools, whatshap, medaka, modkit, tdb."

This should really be a conda yml file or similar - i.e make it simple for users to adopt. Currently this requires a level of bioinformatics ability that is not commonly available.

We thank the reviewer for pointing this out, we have added a environment.yml file for the pipeline. The installation is not simple 3 steps:

micromamba env create -f environment.yml

micromamba activate tbas

pip install -e .

I stopped testing the code at this point.

I was hoping there would be some explanation in the README or elsewhere in the repository of the novelty in the analysis and the underlying assummptions/approach but there is no reference to anythign other than chaining of existing tools.

We thank the reviewer for asking the novelty details, we have updated the README with our novelties.

Reviewer #3 (Remarks to the Author)

I thank the authors for the effort they have made to improve the manuscript.

I prefer the new title and I am happy with how the variant calling / phasing benchmarks are now presented, and how performance in on-target vs off-target regions is reported.

We thank the reviewer for highlighting this and agree that the manuscript has been improved thanks to all reviewers comments.

I feel there is still room for improved clarity in the section on the 13 x trios with rare disease:

1. Please add a section to the Methods to describe the process and criteria by which the 13 cases were selected; what sequencing and or other testing methods had been performed previously; and the extent of previous data analysis. You've provided some of this information in the reviewer rebuttal, but it needs to be added to the main manuscript. Please clarify that the TBAS team/analysts were blind to and previous diagnoses. If selection of cases by the referring clinicians was purely random, please state this.

We thank the reviewer for asking for the details. We have added this paragraph to the method section

Sample selection

All samples in this study were obtained through the GREGoR consortium and had been previously reviewed by consortium clinicians using other established genomic technologies. The only information available to us at the time of analysis was that these cases remained genetically unresolved and their disease phenotypes. Sample selection was restricted to four broad disease categories because the clinician involved in the downstream manual variant inspection has specific expertise in these areas, to ensure that candidate variants could be reviewed by a clinician with relevant disease-area expertise. These disease categories also matched the phenotype-linked panels used for adaptive sampling

Beyond this high-level disease categorization, no additional selection criteria were applied. In particular, there was no prioritization based on phenotype severity, family structure, or prior diagnostic hypotheses. The sole inclusion criterion was that the cases had not been solved by prior clinical evaluation within the consortium. Before TBAS, some cases had been evaluated using narrower phenotype-specific clinical panels. For this study, we used broader phenotype-category panels so that four standardized target sets could cover the selected disease groups.

2. For the 5/8 trio cases that were newly solved using TBAS, there are several instances where good candidate variants identified by TBAS "were later confirmed on reassassement of exome data". Please clarify why those variants were not detected/prioritised during previous analysis. I think it is valuable and important to distinguish these kinds of cases from those where TBAS found a genuinely challenging causative variant that could not be detected with short read data. From what I can gather, all cases apart from the NAXE and possibly the ATN1 repeat expansions, were solvable with existing short-read data. Maybe I'm misinterpreting. Please clarify.

We thank the reviewer for raising this point, and we agree that the distinction should be made clearer. The cases described as newly solved or newly prioritized were unresolved at the time they were submitted for TBAS analysis. In other words, the relevant variant had not been identified as the causative or leading candidate variant in the prior clinical short-read or exome-based analysis. At BCM every trio or proband gets a WES by default up to recently (now it's more often WGS illumina). TBAS and the paired analysis workflow identified these variants as top candidates. After TBAS highlighted their potential relevance, we manually reinspected the available prior Illumina data and variant callsets. This retrospective review showed that the variant was technically present in the short-read data, but had not been prioritized or interpreted as causative before the TBAS analysis.

We do not intend to claim that all newly prioritized cases represent variants that were technically undetectable by short-read sequencing. Rather, TBAS contributed in two ways. For some cases, it identified variant classes where long reads provide a clear technical advantage, including repeat expansions, allele sizing, methylation and inheritance of expanded alleles. For other cases, TBAS provided an independent trio-based long-read dataset and standardized prioritization workflow that elevated variants that had not been recognized in the original short-read clinical analysis. Thus, retrospective detectability in short-read data does not mean that the case was solved before TBAS. It means that TBAS prompted the variant to be recognized and reinterpreted as clinically relevant.

We made this now clearer in the final paragraph in the main text :"The TBAS workflow provides a cost-efficient and comprehensive strategy for phenotype-guided long-read trio analysis in rare disease. Across 13 GREGoR trios, TBAS recovered the known candidate variant in all five previously solved cases and prioritized likely causative or clinically relevant candidates in five of eight previously unresolved cases, resulting in a 76.9% potential solve rate. Across the previously unresolved cases, TBAS contributed in two complementary ways. For repeat expansion cases, especially *NAXE* and *ATN1*, the long-read data enabled direct allele sizing, inheritance assessment and, in the *NAXE* case, methylation support around the expanded allele. In other cases, the TBAS workflow highlighted and reported clinically relevant SNV or indel candidates that had not been prioritized during earlier clinical review. Retrospective inspection of prior short-read or exome data showed that some of these variants were technically present, but had not been recognized as leading candidates before TBAS analysis. Thus, the added value of TBAS was not limited to detecting variants absent from short-read data. It also provided independent trio-based prioritization and molecular interpretation across variant classes."

If these points can be addressed then I would be supportive of publication.

I'd also point the reviewers to two similar studies I was involved with, where we used barcoded ONT adaptive sampling to improve rare disease diagnosis (2-4 patients per flow cell, though not specifically focused on family trios like TBAS), which might optionally be cited. DOIs:

10.1002/acn3.70008

10.64898/2025.12.06.25340828

We thank the reviewer for the suggestions, these 2 papers are indeed relevant, and we have cited them in our manuscript now.

All the best

Ira Deveson

(Remarks on code availability)

I concur with Reviewer 1's comments that the code here should not be described as novel tool or software package. The code provided is appropriate to document/reproduce the method followed for this study, but it is not a generic, easily deployable package that others could easily adopt, especially in a clinical context.

We thank the reviewer for the comment, along with the reviewer 1's point, we have utilized conda / micromamba to it is easy to be deployed with only 3 commands:

micromamba env create -f environment.yml

micromamba activate tbas

pip install -e .
